# Supplementary material for: Exploring high-resolution cryo-ET and subtomogram averaging capabilities of contemporary DEDs
Source: J Struct Biol. Author manuscript; Available in PMC 2025 Mar 17. (PMC11912802; doi:10.1016/j.jsb.2022.107852)
Supplement: Supplemental Figures [file NIHMS1816855-supplement-Supplemental_Figures.docx]

**Supplementary Information for**

**Exploring high-resolution cryo-ET and subtomogram averaging capabilities of** **contemporary DEDs**

Martin Obr^1^, Wim JH Hagen^2^, Robert A Dick^3^, Lingbo Yu^4^, Abhay Kotecha^4^, Florian KM Schur^1*^

^1^ Institute of Science and Technology Austria, Klosterneuburg, Austria

^2^ European Molecular Biology Laboratory (EMBL) Heidelberg, Germany

^3^ Department of Molecular Biology and Genetics, Cornell University, Ithaca, USA

^4^ Materials and Structural Analysis Division, Thermo Fisher Scientific, Eindhoven,

Netherlands

^*^ Corresponding author: florian.schur@ist.ac.at

**Supplementary Figures**


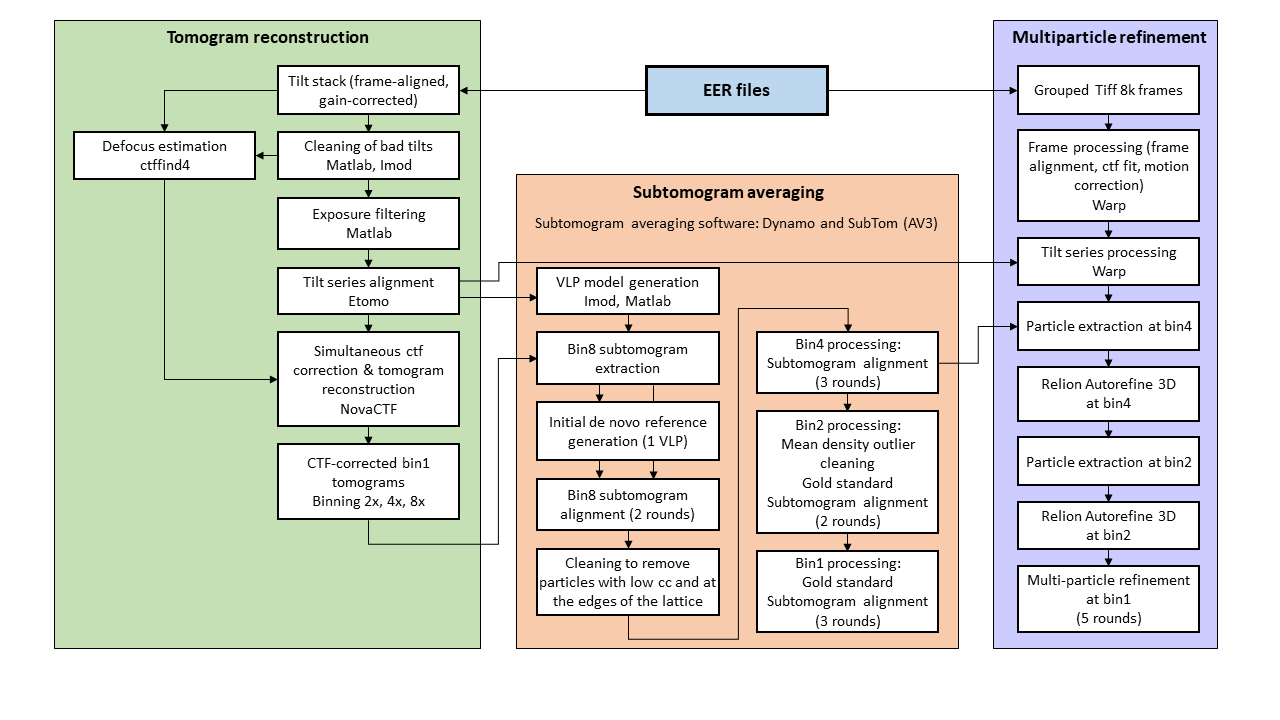


**Figure S1: Data processing chart** Diagram of the image processing workflows employed in this study. The green sector indicates pre-processing and tomogram reconstruction, the orange sector contains subtomogram averaging performed in MATLAB, Dynamo, and subTOM (AV3); and the purple sector contains all steps performed in Relion and M.


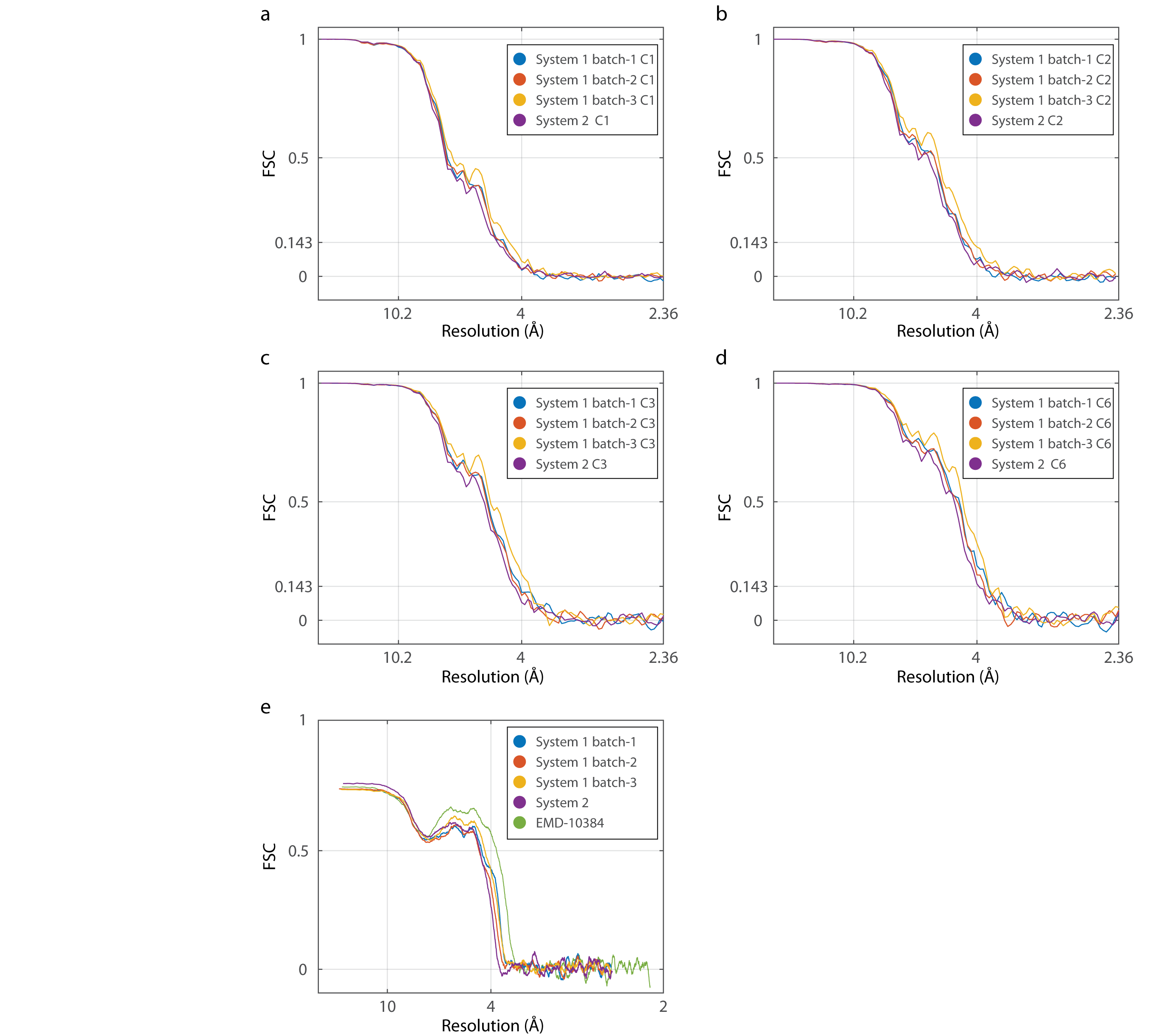


**Figure S2 Fourier shell correlation of symmetrized maps solved using System 1 and System 2, corresponding to data shown in Figure 2. a-d** FSCs of symmetrized maps **a** – C1, **b** – C2, **c** – C3, and **d** – C6. The FSC crossings at 0.5 and 0.143 correspond to Figure 2 and the values in Supplementary Table 2. **e** model vs. map FSC using pdb 6T64, which was refined into into a cryo-ET map of EIAV CASP solved at 3.7 Å (EMD-10384).

**
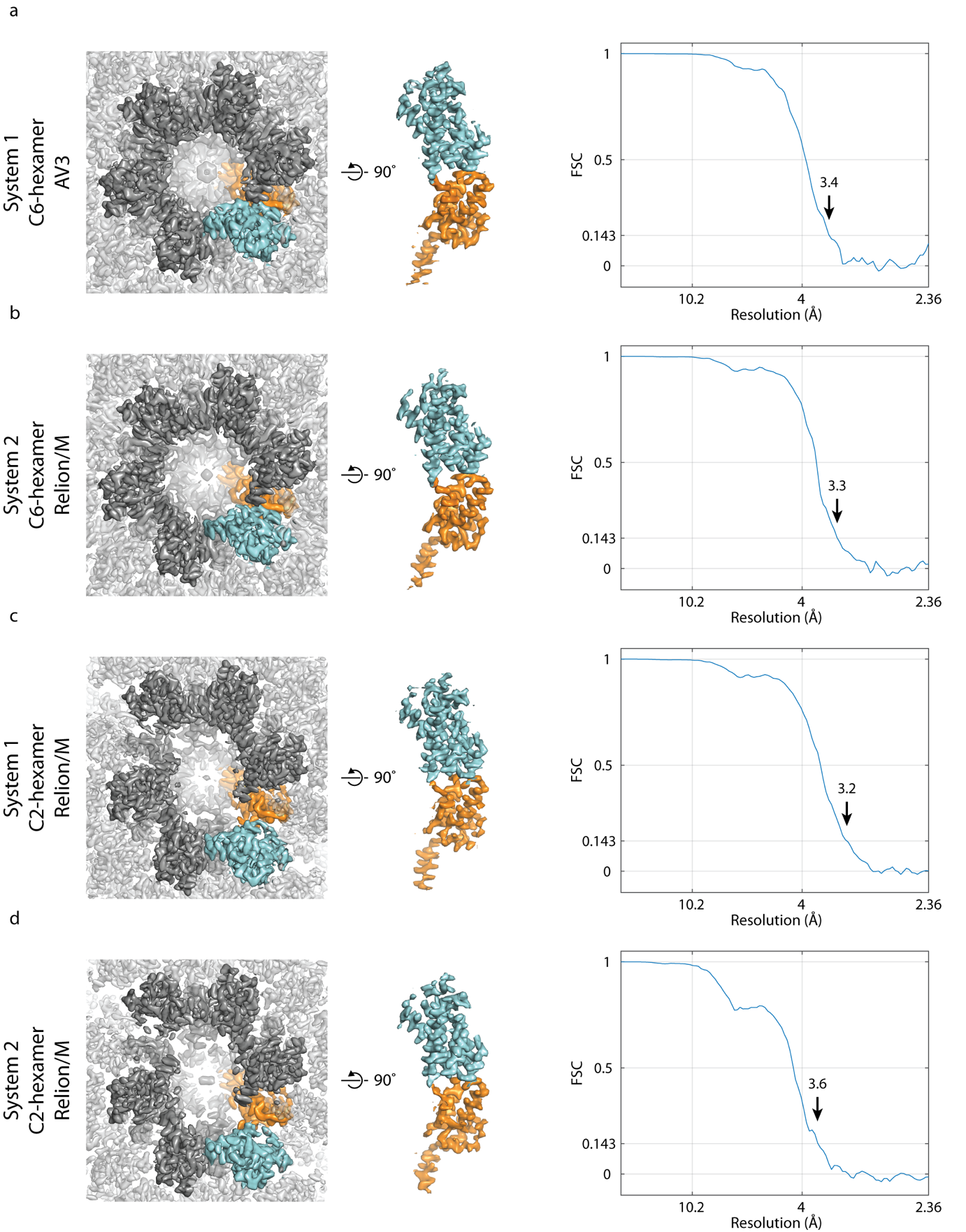
**

**Figure S3: Cryo-ET maps solved by STA using data from System 1 and System 2. a-d)** Isosurface representations of sharpened cryo-ET maps, generated either via the NovaCTF/AV3-based or Relion/M-based workflows. The origin of the data (System 1 or 2), the respective average (C6-hexamer from spherical VLPs, or C2-hexamer from tubular VLPs), and the used software are shown at the left. The central CA hexamer of the solved structures is shown in dark gray, with one CA monomer within the hexamer highlighted with cyan, and orange colors for the NTD and CTD, respectively. The corresponding FSC-curves are shown on the right. **a)** EIAV C6-symmetric CA hexamer solved by the NovaCTF/AV3 pipeline from the full System 1 dataset. The obtained resolution at the 0.143 FSC criterion is 3.4Å. **b)** EIAV C6-symmetric CA hexamer solved by Multiparticle refinement from the full System 2 dataset**.** The obtained resolution at the 0.143 FSC criterion is 3.3Å. **c)** EIAV C2-symmetric CA hexamer solved by Multiparticle refinement from the full System 1 dataset. The obtained resolution at the 0.143 FSC criterion is 3.2Å. **d)** EIAV C2-symmetric CA hexamer solved by Multiparticle refinement from the full System 2 dataset**.** The obtained resolution at the 0.143 FSC criterion is 3.6Å.

**
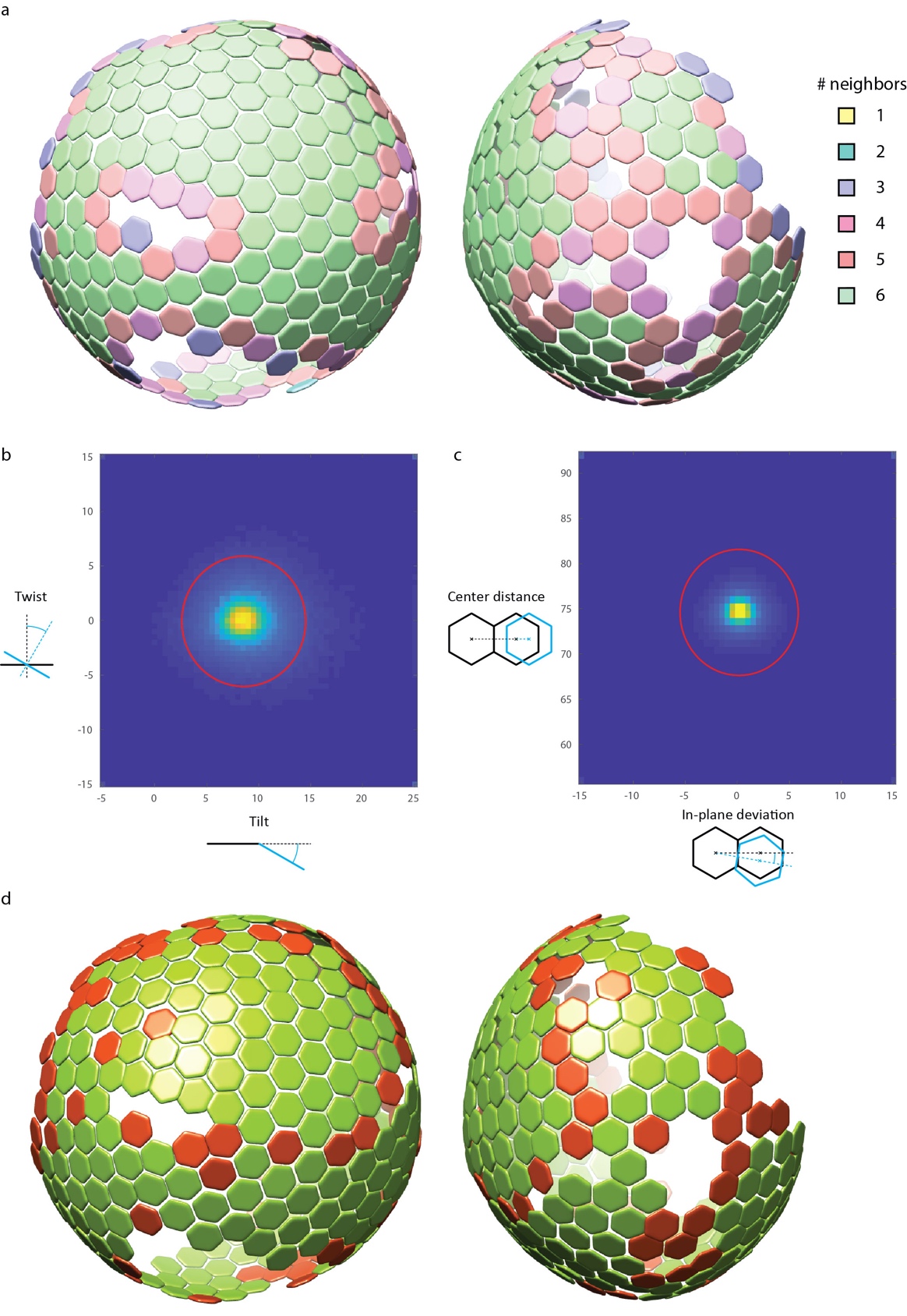
**

**Figure S4: Geometry cleaning of the EIAV immature CANC lattice.** **a)** Schematic representation of hexamer positions for a given VLP. Color code corresponds to the number of neighboring hexamers: green – 6; salmon – 5; pink – 4; purple – 3; light blue – 2; yellow – 1. **b)** A plot visualizing the tilt and twist angles for the individual hexamer-hexamer neighbors. The red circle shows the threshold used for cleaning. **c)** A plot visualizing the in-plane angle deviation and the center-to-center distance for the individual hexamer-hexamer neighbors. The red circle shows the threshold used for the cleaning. **d**) Same as **a**, shown in green positions that fulfill conditions visualized in **b** and **c** for at least 3 neighbors; the remaining outlier positions are shown in red.


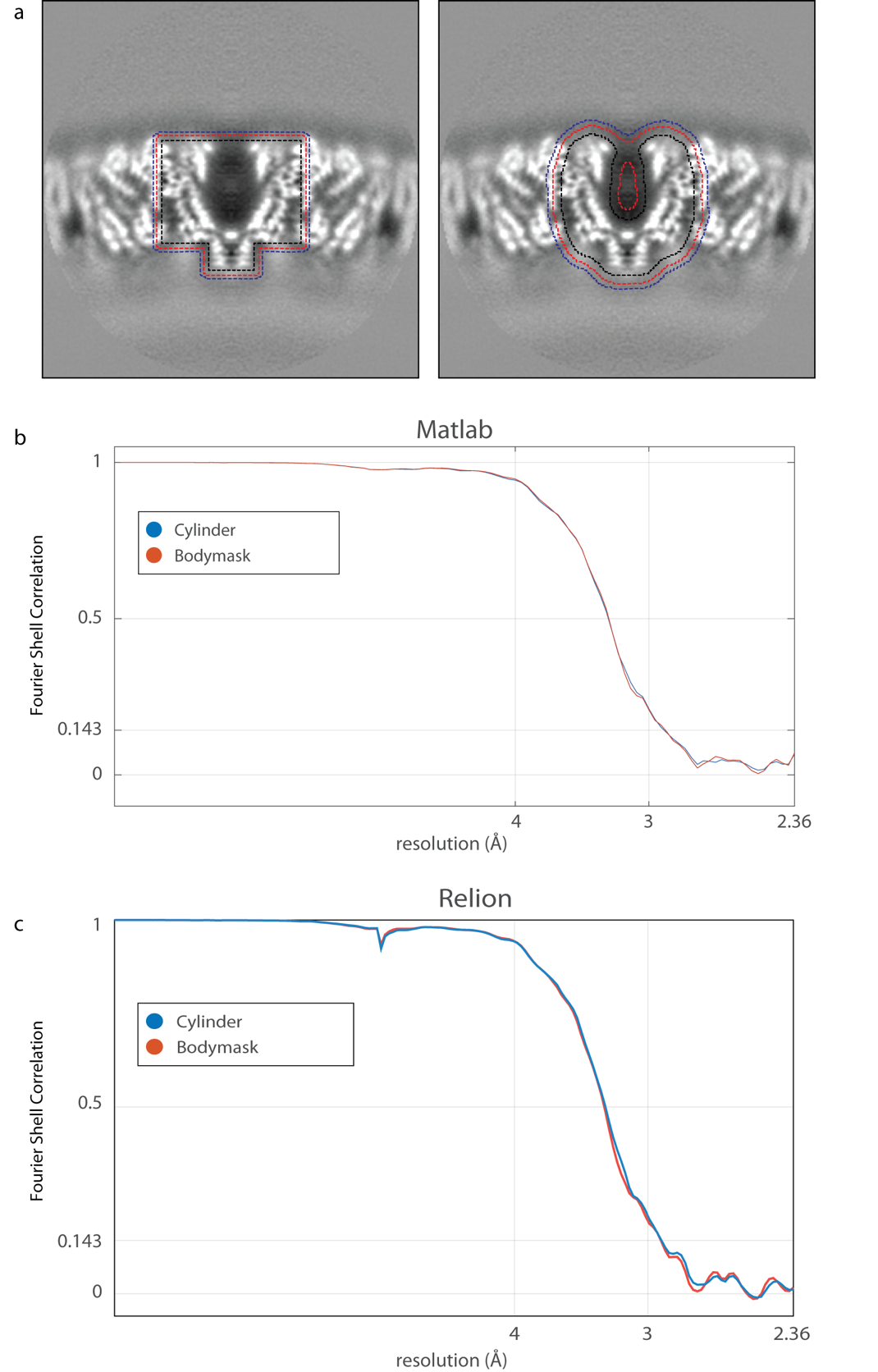


**Figure S5: Effects of the mask on the FSC.** **a**) Central slice through the C6-symmetric map obtained by Multiparticle refinement for System 1. The boundary of the masks, either cylindrical or body-shaped is shown by dotted lines. Black dotted line –isosurface threshold at 1.0, red dotted line – isosurface threshold at 0.67, and blue dotted line – isosurface threshold at 0.33. **b)** Phase-randomized and masked FSC performed using MATLAB. **c** Phase-randomized, masked FSC in Relion 3.0.7.

|  |  | System 1 | System 2 |
| --- | --- | --- | --- |
| Dataset |  | EIAV CANC Falcon 4 | EIAV CANC K3 |
| Acquisition settings | **Microscope** | TFS Titan Krios G4 | TFS Titan Krios G3i |
|  | **Voltage (keV)** | 300 | 300 |
|  | **Electron source** | E-CFEG | XFEG |
|  | **Detector** | TFS Falcon 4 | Gatan K3 |
|  | **Energy-filter** | TFS Selectris X | Gatan BioQuantum |
|  | **Slit width (eV)** | 10 | 20 |
|  | **Super-resolution**  **Mode** | Yes | Yes |
|  | **Å/ physical pixel** | 1.179 | 0.822 |
|  | **Defocus range**  **(µm)** | -0.75 to 3.25 | -0.75 to 3.25 |
|  | **Defocus step**  **(µm)** | 0.25 | 0.25 |
|  | **Acquisition scheme** | -60/60°, 3°  Dose-symmetric  Tomography 5.0 | -60/60°, 3°  Dose-symmetric  SerialEM |
|  | **Total dose**  **(electrons/ Å^2^)** | ~143.5 | ~146 |
|  | **Dose rate**  **(electrons/ Å^2^/sec)** | 3.43 | 38.2 |
|  | **File Format** | EER  (electron event representation) | tif  (hardware summed raw frames) |
|  | **Frame number** | Not applicable | 7 |
|  | **Tilt series used for STA** | 85 (84)^*^ | 35 |

**Supplementary Table 1: Acquisition settings**

^*^ - 85 tilt series were used in the AV3 alignment, but only 84 were subjected to processing in Relion/M.

| System | Dataset | Symmetry | Particle number | (Sub-) tomogram reconstruction | STA Processing | FSC 0.143 (Å) | Fourier shells above 0.143 crit. |
| --- | --- | --- | --- | --- | --- | --- | --- |
| 1 |  |  |  |  |  |  |  |
|  | batch 1 | C6 | 20 000 | novaCTF | AV3 | 3.8 | 69/112 |
|  | batch 2 | C6 | 20 000 | novaCTF | AV3 | 3.9 | 68/112 |
|  | batch 3 | C6 | 20 000 | novaCTF | AV3 | 3.8 | 69/112 |
|  | full dataset | C6 | 62 282 | novaCTF | AV3 | 3.4 | 78/112 |
|  | full dataset | C6 | 77 659 | Warp | Relion, Multiparticle refinent | 2.9 | 136/170 |
|  | full dataset | C2 | 53 665 | Warp | Relion, Multiparticle refinement | 3.2 | 125/170 |
| 2 |  |  |  |  |  |  |  |
|  | batch 1 | C6 | 20 000 | novaCTF | AV3 | 3.9 | 66/112 |
|  | full dataset | C6 | 32 446 | Warp | Relion, Multiparticle refinement | 3.3 | 121/174 |
|  | full dataset | C2 | 18 596 | Warp | Relion, Multiparticle refinement | 3.6 | 112/174 |

**Supplementary Table 2: Dataset overview**

| SYSTEM 1 | | | | | | |
| --- | --- | --- | --- | --- | --- | --- |
| Iteration | Image warp | Volume warp | Poses | Defocus | Stage angles | Movie refinement |
| 1 | 4x4 | 2x2x1x10 | N | N | N | N |
| 2 | 4x4 | 2x2x1x10 | Y | N | N | N |
| 3 | 8x8 | 4x4x2x20 | Y | Grid search | N | N |
| 4 | 8x8 | 4x4x2x20 | Y | Y | Y | Y |
| 5 | 8x8 | 4x4x2x20 | Y | Y | Y | Y |

**Supplementary Table 3: Multiparticle refinement parameters for System 1 dataset**

| SYSTEM 2 | | | | | | |
| --- | --- | --- | --- | --- | --- | --- |
| Iteration | Image warp | Volume warp | Poses | Defocus | Stage angles | Movie refinement |
| 1 | 4x3 | 2x2x1x10 | N | N | N | N |
| 2 | 4x3 | 2x2x1x10 | Y | N | N | N |
| 3 | 8x6 | 3x4x2x20 | Y | Grid search | N | N |
| 4 | 8x6 | 3x4x2x20 | Y | Y | Y | Y |
| 5 | 8x6 | 3x4x2x20 | Y | Y | Y | Y |

**Supplementary Table 4: Multiparticle refinement parameters for System 2 dataset**
